# Consistency of dark skeletal muscles in Thai native black-bone chickens (*Gallus gallus domesticus*)

**DOI:** 10.7717/peerj.10728

**Published:** 2021-01-13

**Authors:** Wannapimol Kriangwanich, Promporn Piboon, Wirakorn Sakorn, Kittisak Buddhachat, Varankpicha Kochagul, Kidsadagon Pringproa, Supamit Mekchay, Korakot Nganvongpanit

**Affiliations:** 1 Department of Veterinary Biosciences and Public Health, Faculty of Veterinary Medicine, Chiang Mai University, Chiang Mai, Thailand; 2 Department of Biology, Faculty of Science, Naresuan University, Phitsanulok, Thailand; 3 Excellence Center in Veterinary Bioscience, Chiang Mai University, Chiang Mai, Thailand; 4 Veterinary Diagnostic Laboratory, Faculty of Veterinary Medicine, Chiang Mai University, Chiang Mai, Thailand; 5 Department of Animal and Aquatic Sciences, Faculty of Agriculture, Chiang Mai University, Chiang Mai, Thailand

**Keywords:** Avian, Black-bone, Chicken, Meat, Muscle

## Abstract

Black-bone chickens (*Gallus gallus domesticus*) have become economically valuable, particularly in Southeast Asia as a consequence of popular traditional Chinese medical practices. Chickens with whole body organ darkness are considered to have higher value and are, therefore, more often requested. This research study aimed to investigate the darkness in 34 skeletal muscles of 10 Thai black-bone chickens (five males and five females). The evaluation of muscle darkness was done on two levels: (i) a color chart was employed at the macroanatomical level and (ii) by using melanin pigment to evaluate the structure at the microanatomy level. The results revealed that the accumulation of melanin pigment in the muscle tissue was observed in the endomysium, perimysium and epimysium. With respect to the results of the color chart test, iliotibialis lateralis pars preacetabularis, gastrocnemius, fibularis longus and puboischiofemoralis pars medialis showed the highest degree of darkness, while serratus profundus, pectoralis, iliotibialis cranialis, flexor cruris lateralis, and flexor cruris medialis appeared to be the least dark. In addition, we found that the highest and lowest amounts of melanin pigment was noted in the flexor carpi ulnaris and pectoralis (*p* < 0.05), respectively; however, there was no significant difference (*p* > 0.05) observed between the sexes. These results reveal that the 34 specified muscles of black-bone chickens showed uneven distribution of darkness due to the differing accumulations of melanin pigments of each muscle.This information may provide background knowledge for a better understanding of melanin accumulation and lead to breeding improvements in Thai black-bone chickens.

## Introduction

Presently, more than 25 breeds of chickens with internal organ hyperpigmentation (hypermelanosis) have been identified to have originated from Southeast Asia (Dorshorst et al., 2011). One well-known breed of black-bone chicken is the Silky fowl (*Gallus gallus domesticus* Brisson), which originated from the southern region of China (Tian et al., 2011). On the other hand, many of the other black-bone chicken breeds also originated from China, such as the Dehua black-bone chicken, the Jiangshan black-bone chicken, the Jinhu black-bone chicken, the Lueyang black-bone chicken, the Muchuan black-bone chicken, the Sichuan black-bone chicken, the Wumeng black-bone chicken, the Xingwen black-bone chicken, the Yanjin black-bone chicken and the Yugan black-bone chicken (Zhu et al., 2014). Presently, black-bone chickens have become economically valuable, particularly in Southeast Asian countries as a consequence of popular traditional Chinese medical practices and for their antioxidant contents and amino acid/peptide profiles, for example, carnosine (Dorshorst et al., 2011; Lukanov & Genchev, 2013; Tian et al., 2007; Tu et al., 2009). Moreover, the meat obtained from black-bone chickens is reported to contain lower amounts of fat and cholesterol than the meat of white chickens (Jaturasitha et al., 2008; Tian et al., 2011).

Black-bone chickens are known for their special phenotypes and the atypical distribution of melanocytes among the internal organs. The migratory path of melanoblasts and premelanocytes and the identity of the genes that are involved in migration during embryogenesis are well-known (Han et al., 2015; Kawakami & Fisher, 2011; Lukanov & Genchev, 2013; Ortolani-Machado et al., 2008). However, the pattern of hyperpigmentation in the organs is different among differing animal species for reasons that are still unknown. For consumer purposes, hyperpigmentations in all organs are highly requested by customers. This has forced sellers to select only black-bone chickens with all black organs. In particular, the muscles should be entirely black. A previous study has shown that the melanin content is different among the tissues/organs of black-bone chickens (Chen et al., 2008; Muroya et al., 2000). In 2000, a measurement of the pigment content using infrared (IR) spectrum revealed that the melanin in the periosteum > gonads (ovary or testis) = trachea ≥ heart, liver, gizzard, cecum, muscles (pectoralis and supracoracoideus) and skin (Muroya et al., 2000). In 2008, scientists reported that the yields of pigment obtained from the periosteum, ovary or testis, trachea, skin and muscles were 21.3, 13.7, 10.2, 1.1 and 1.0 mg/g, respectively by using X-ray photoelectron spectroscopy (XPS) technique (Chen et al., 2008).

Thai black-bone chickens are indigenous strains to Thailand, where they are usually reared in rural and mountainous areas. The dark bones in the Thai black-bone chickens are another special feature that are favored by certain consumers. Additionally, the meat of the chicken must be darker than that of normal chickens (Jaturasitha et al., 2008). However, the degree of darkness in the organs of Thai black-bone chickens is usually not consistent, especially in the muscles which can show variations from darkness to brightness. Chickens with brighter muscles usually have less value than chickens with darker muscles, and this is also true with the other organs. Therefore, breeding improvements are needed in terms of acquiring better control of the consistency of the degree of darkness in the muscles and organs of these species of chickens. However, little is known about the patterns of muscle darkness in the black-bone chicken. The objective of this research study was to investigate the consistency of muscle darkness in Thai black-bone chickens. We have hypothesized that the darkness of the muscle in Thai black-bone chickens was found to be inconsistent. This study employed two methods that included macroanatomy and histological studies. In the macroanatomy study, the darkness of the muscle is described, while the location and density of the melanin pigment that is accumulated in the muscle fiber is described in the histological study.

## Materials and Methods

### Animals

**Figure 1:**
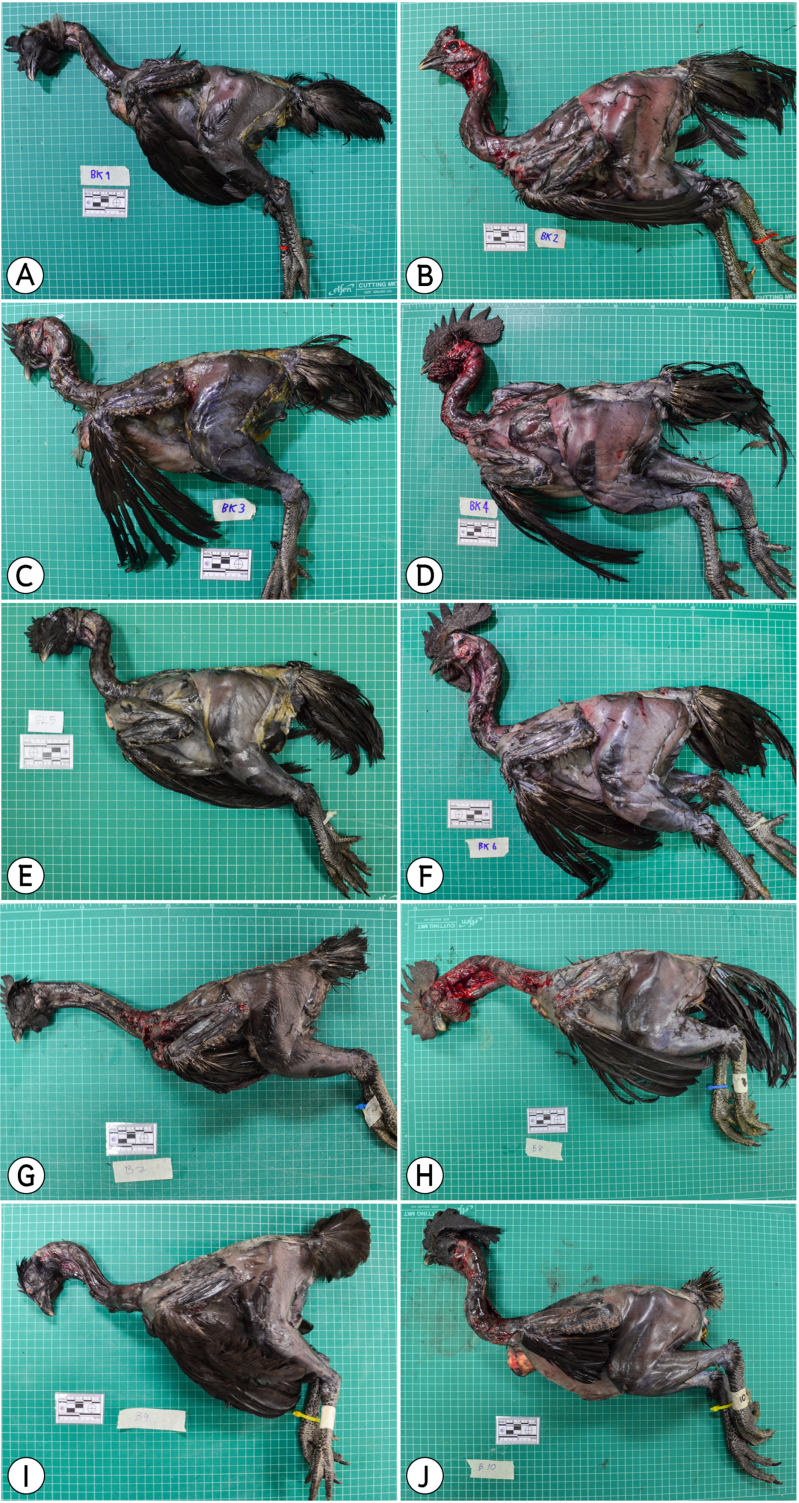
Ten cadavers of black-bone chickens used in this study; (A) (C6), (C) (C7), (E) (C8), (G) (C9), (I) (C10) were female, (B) (C1), (D) (C2), (F) (C3), (H) (C4), (J) (C5) were male.

Ten adult cadavers of black-bone chickens (age 8 months, male = 5; C1–C5, female = 5; C6–C10) were obtained from the Royal Project, Chiang Mai, Thailand (Fig. 1). These 10 chickens were fed in the same hatchery. In total, 34 muscles with diameters larger than 0.5 cm^3^ were use as subjects in this study (Table 1). The Animal Ethics Committee, Faculty of Veterinary Medicine, Chiang Mai University approved this research animal used by the license number S23/2561.

**Table 1:** List of 34 muscles investigated in this study.

No.	Muscle name	Body part
1	Latissimus dorsi	Pectoral girdle
2	Serratus profundus	Pectoral girdle
3	Pectoralis	Pectoral girdle
4	Obliquus externus abdominis	Abdominal wall
5	Complexus	Vertebral column
6	Biventer cervicis	Vertebral column
7	Longus colli ventralis	Vertebral column
8	Deltoidius pars propatagialis	Wing
9	Extensor carpi radialis	Wing
10	Ectepicondyloulnaris	Wing
11	Extensor carpi ulnaris	Wing
12	Deltoidius pars major	Wing
13	Triceps brachii	Wing
14	Biceps brachii	Wing
15	Flexor carpi ulnaris	Wing
16	Flexor digitorum profundus	Wing
17	Pronator superficialis	Wing
18	Iliotibialis cranialis	Pelvic limb
19	Iliotibialis lateralis pars preacetabularis	Pelvic limb
20	Iliotibialis lateralis pars postacetabularis	Pelvic limb
21	Flexor cruris lateralis	Pelvic limb
22	Gastrocnemius	Pelvic limb
23	Flexor perforans et perforatus digiti II	Pelvic limb
24	Flexor perforans et perforatus digiti III	Pelvic limb
25	Fibularis longus	Pelvic limb
26	Iliotrochantericus cranialis	Pelvic limb
27	Iliotrochantericus medius	Pelvic limb
28	Iliofemorarlis internus	Pelvic limb
29	Femorotibailis lateralis	Pelvic limb
30	Flexor cruris medialis	Pelvic limb
31	Puboischiofemoralis pars medialis	Pelvic limb
32	Femorotibailis medialis	Pelvic limb
33	Ambiens	Pelvic limb
34	Femorotibialis intermedius	Pelvic limb

### Assessment of data and statistical analysis

#### Macroanatomy and color grading via color chart

**Figure 2:**
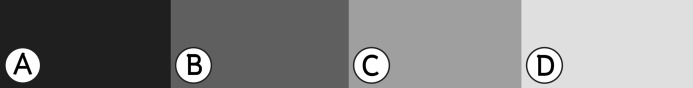
Color chart used in this study. Black: #1f1f1f (A), Dim gray: #5f5f5f (B), Dark gray: #9f9f9f (C) and Silver: #dfdfdf (D).

The macroanatomy of muscles was observed and a color chart (Fig. 2) was used for grading by two investigators within 6 h after the animals had died. The muscles were scored on both sides of the body. A four-color chart was designed by using the quartile system. Using the color chart in Photoshop Version 7 (Adobe), 256 color levels were identified from black (#000000) to white (#ffffff). Black on the color chart (#1f1f1f) ranged from #000000 to #3f3f3f. Dim gray (#5f5f5f) on the color chart ranged from #404040 to #7f7f7f. Dark gray (#9f9f9f) on the color chart ranged from #808080 to # bfbfbf. Lastly, silver (#dfdfdf) on the color chart ranged from #c0c0c0 to #ffffff.

#### Histological study

The histological technique was employed as previous described in Nganvongpanit et al. (2020) and Thitaram et al. (2018). First, tissue samples were fixed in 10% neutral buffered formalin and decalcified with 10% nitric acid. The specimens were cut and placed in plastic cassettes and then processed into the paraffin block and cut into 4 µm sections. Sections were deparaffinized and rehydrated then stained with Harris’s hematoxylin and 1% eosin Y. Graded ethanol series were used for dehydrating sections and then, cleared in xylene and mounted in Permount.

Three places in each muscle of each chicken were chosen randomly and cut into pieces for preparing three slides. In each muscle of each chicken, the muscle was randomly cut in three places to produce three pieces. Each piece of tissue was use to prepare three slides. Compound light microscope (Olympus BX53, Tokyo, Japan) with AxioVision 4.8.2 software (Carl Zeiss, Berlin, Germany) was used to observe individual section. For each slide, all areas of each tissue specimen were recorded to evaluate the percentage of melanin pigment. The area of the melanin pigment and tissue in each muscle was used to calculate the overall percentage of melanin pigment. The percentage of melanin pigment was calculated from 100*(area of melanin pigment/are of tissue). Other characteristics of the melanin pigment, such as the location of presenting, were also recorded.

### Statistical analysis

Data were expressed as mean ± standard deviations (SD). The mean values of color of each muscle according to the grading scale of the color chart were compared using a clustered heat map (a combined of dendrograms and heatmap) (Nganvongpanit et al., 2020; Ryan et al., 2020). We colored the cells accorded to the percentage of melanin pigment, where the highest melanin pigment is represented by the color black and the lowest melanin pigment is presented in silver. Data on the macroscopic and microscopic examinations and the patterns of the melanin pigment are presented using descriptive statistics. The percentage of melanin pigment among the muscles was compared using one-way ANOVA. However, if ANOVA assumptions were not met, we employed the Wilcoxon test. Additionally, differences in the mean values of percentage of melanin pigment between sexes were compared using the Student’s *T*-test. *P*-values of <0.05 were considered statistically significant. All statistical analyses and the dendrogram generation were performed using R version 3.6.3 (R Core Team, 2013).

## Results

### Macroanatomy color grading via color chart

**Figure 3:**
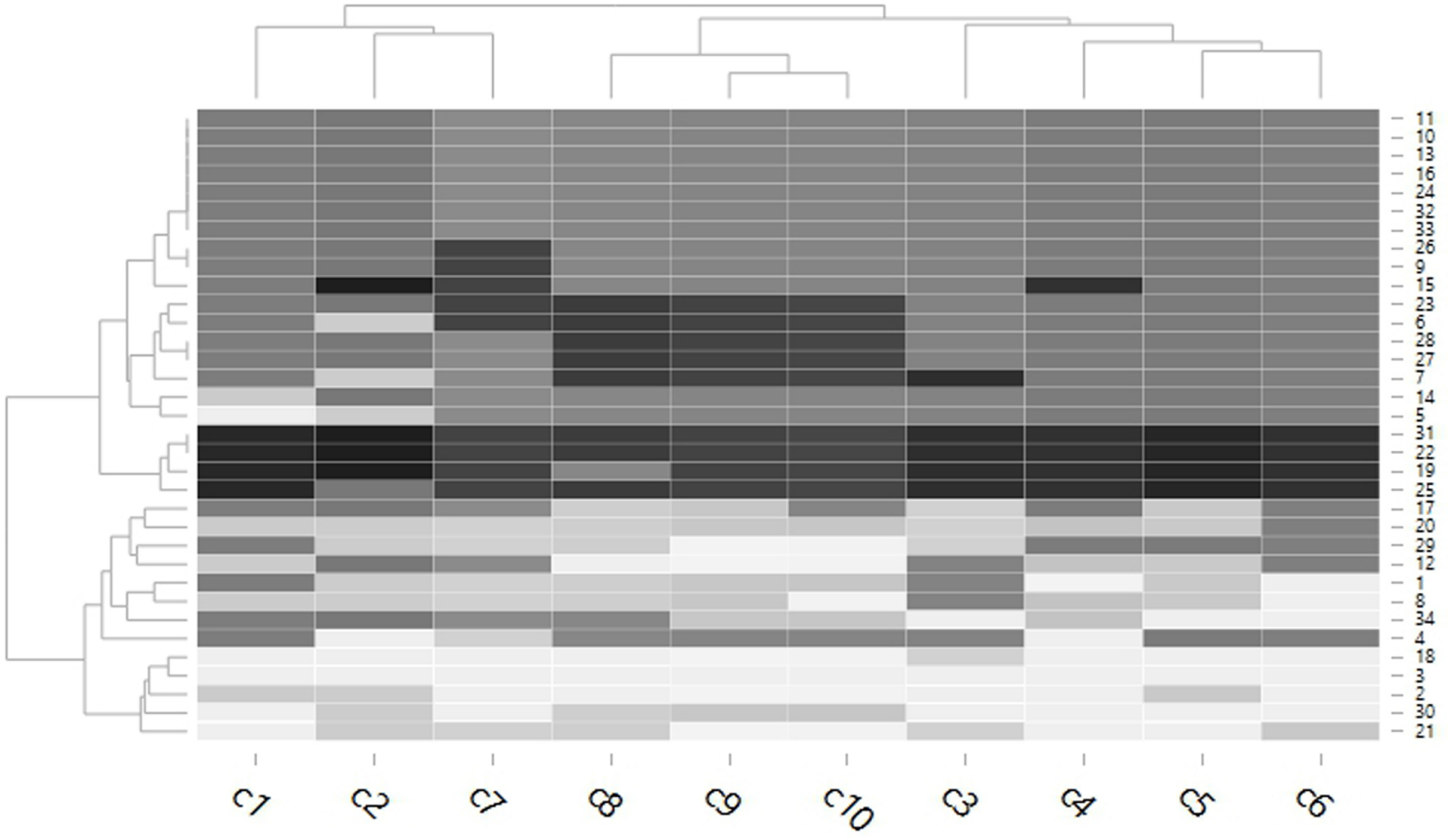
Dendrogram of the color chart mean scored grading of each muscle in each chicken. The cells are colored according to the mean of color chart grading score. The highest darkness is represented by the color black, while the lowest darkness is presented in super light gray. A tree on the column represents a clustered group of chickens (C1–C5 were male, C6–C19 were female), while a tree on the row represents a clustered group of muscles. 1 = latissimus dorsi, 2 = serratus profundus, 3 = pectoralis, 4 = obliquus externus abdominis, 5 = complexus, 6 = biventer cervicis, 7 = longus colli ventralis, 8 = deltoidius pars propatagialis, 9 = extensor carpi radialis, 10 = ectepicondyloulnaris, 11 = extensor carpi ulnaris, 12 = deltoidius pars major, 13 = triceps brachii, 14 = biceps brachii, 15 = flexor carpi ulnaris, 16 = flexor digitorum profundus, 17 = pronator superficialis, 18 = iliotibialis cranialis, 19 = iliotibialis lateralis pars preacetabularis, 20 = iliotibialis lateralis pars postacetabularis, 21 = flexor cruris lateralis, 22 = gastrocnemius, 23 = flexor perforans et perforatus digiti II, 24 = flexor perforans et perforatus digiti III, 25 = fibularis longus, 26 = iliotrochantericus cranialis, 27 = iliotrochantericus medius, 28 = iliofemorarlis internus, 29 = femorotibailis lateralis, 30 = flexor cruris medialis,31 = puboischiofemoralis pars medialis, 32 = femorotibailis medialis, 33 = ambiens, 34 = femorotibialis intermedius.

Figure 3 showed the color chart represented the clustering of 2 main groups of 21 dark colored muscles and 13 light colored muscles by dendrogram. The dark muscles with the highest degree of darkness included iliotibialis lateralis pars preacetabularis, gastrocnemius, fibularis longus and puboischiofemoralis pars medialis. The least dark muscles included serratus profundus, pectoralis, iliotibialis cranialis, flexor cruris lateralis, and flexor cruris medialis. Additionally, the tree on the column could represent clustering into two main groups and it was determined that gender did not have as effect on the overall variation in the darkness of muscle.

### Histological evaluation

**Figure 4:**
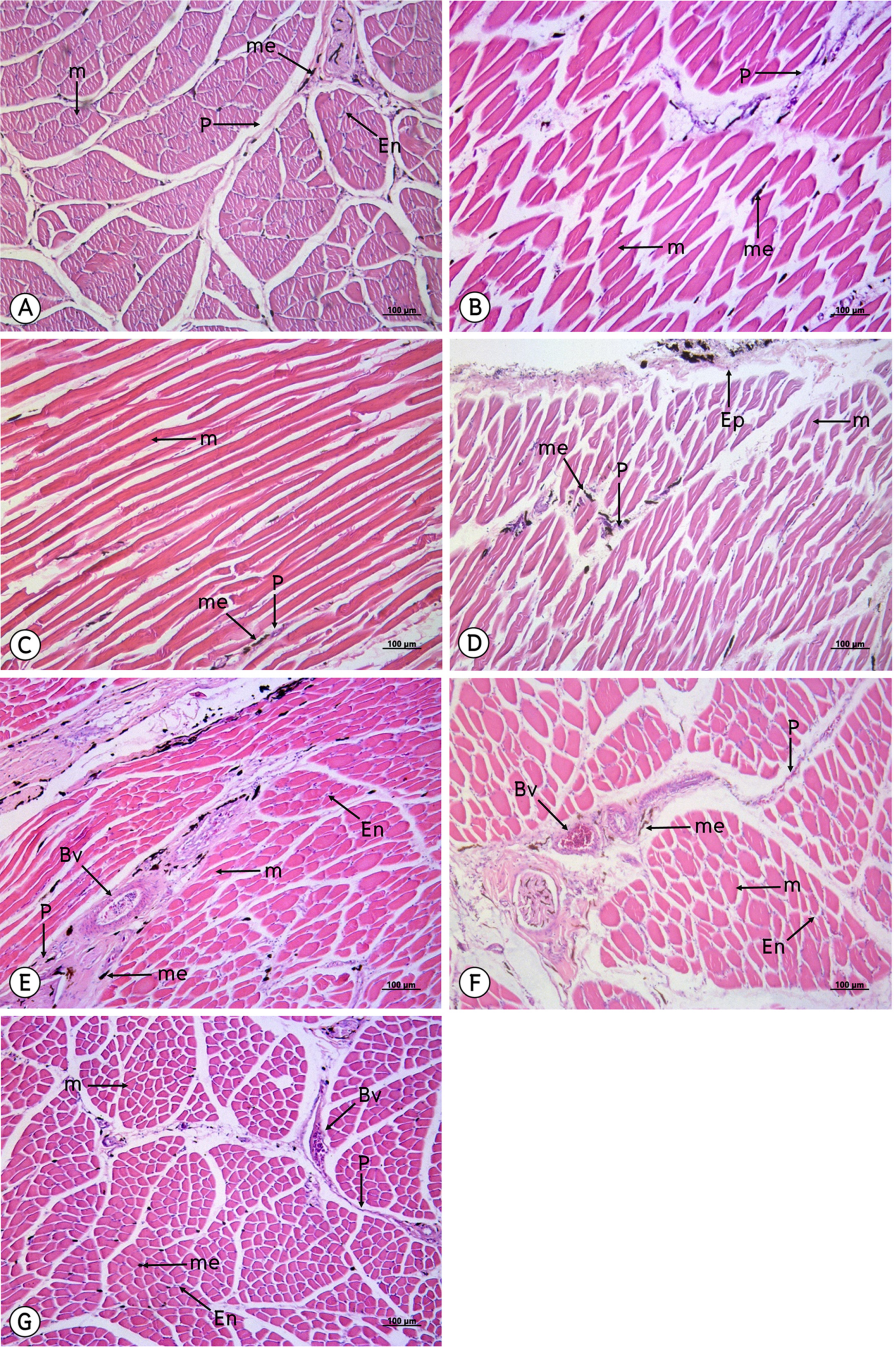
Low magnification of the histological results of striated muscles from the latissimus dorsi (A), serratus profundus (B), pectoralis (C), obliquus externus abdominis (D), complexus (E), biventer cervicis (F) and longus colli ventralis (G). Melanin pigment accumulated in the epimysium, perimysium and endomysium, but did not accumulate in the muscle fiber. Abbreviations: Bv, blood vessel; En, endomysium; M, muscle fiber; me, melanin pigment; P, perimysium. Hematoxylin and eosin staining.

**Figure 5:**
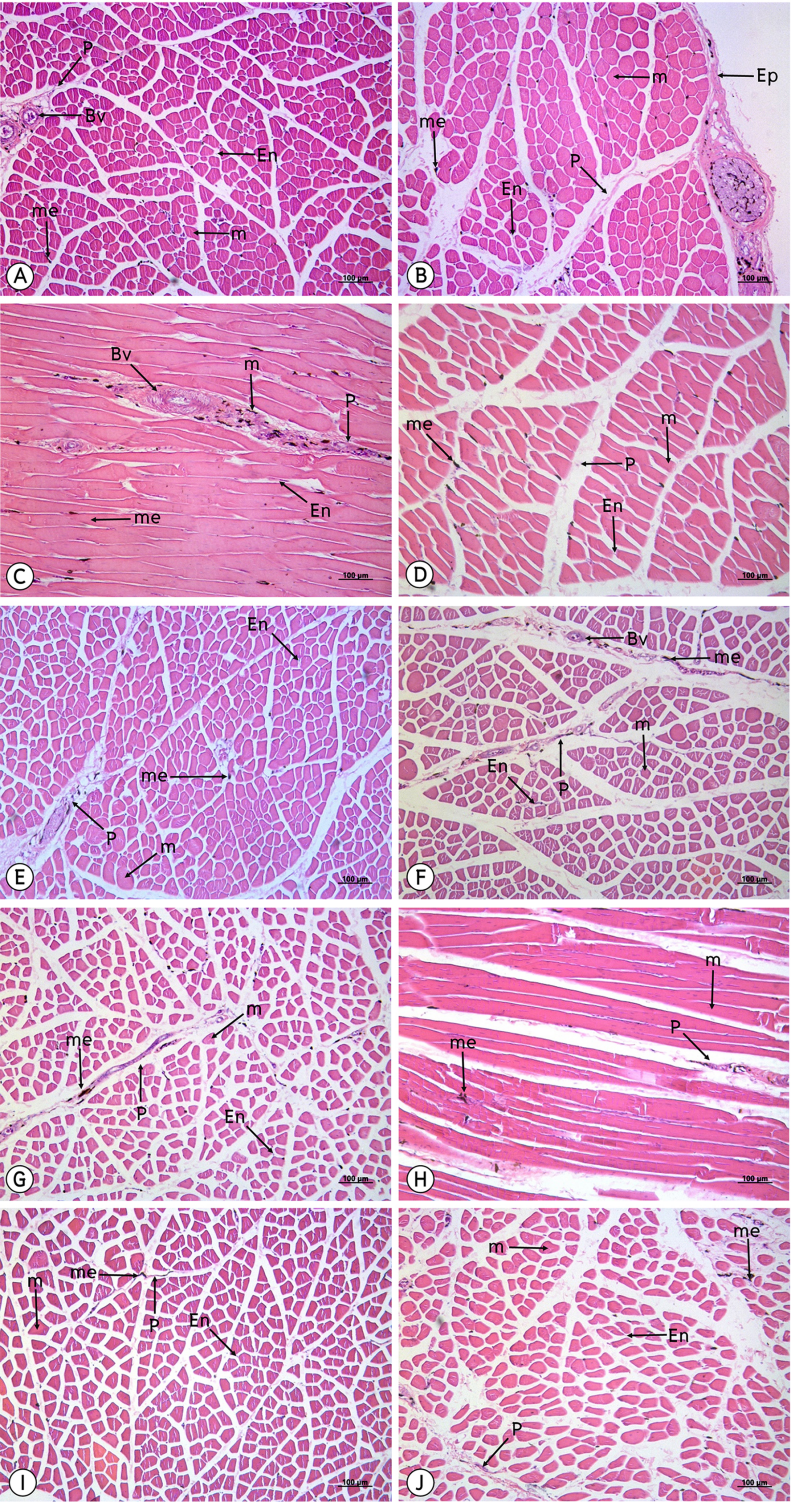
Low magnification of histological results of striated muscles from deltoidius pars propatagialis (A), extensor carpi radialis (B), ectepicondyloulnaris (C), extensor carpi ulnaris (D), deltoidius pars major (E), triceps brachii (F), biceps brachii (G), flexor carpi ulnaris (H), flexor digitorum profundus (I) and pronator superficialis (J). Melanin pigment accumulated in the epimysium, perimysium and endomysium, but did not accumulate in the muscle fiber. Abbreviations: Bv, blood vessel; En, endomysium; M, muscle fiber; me, melanin pigment; P, perimysium. Hematoxylin and eosin staining.

**Figure 6:**
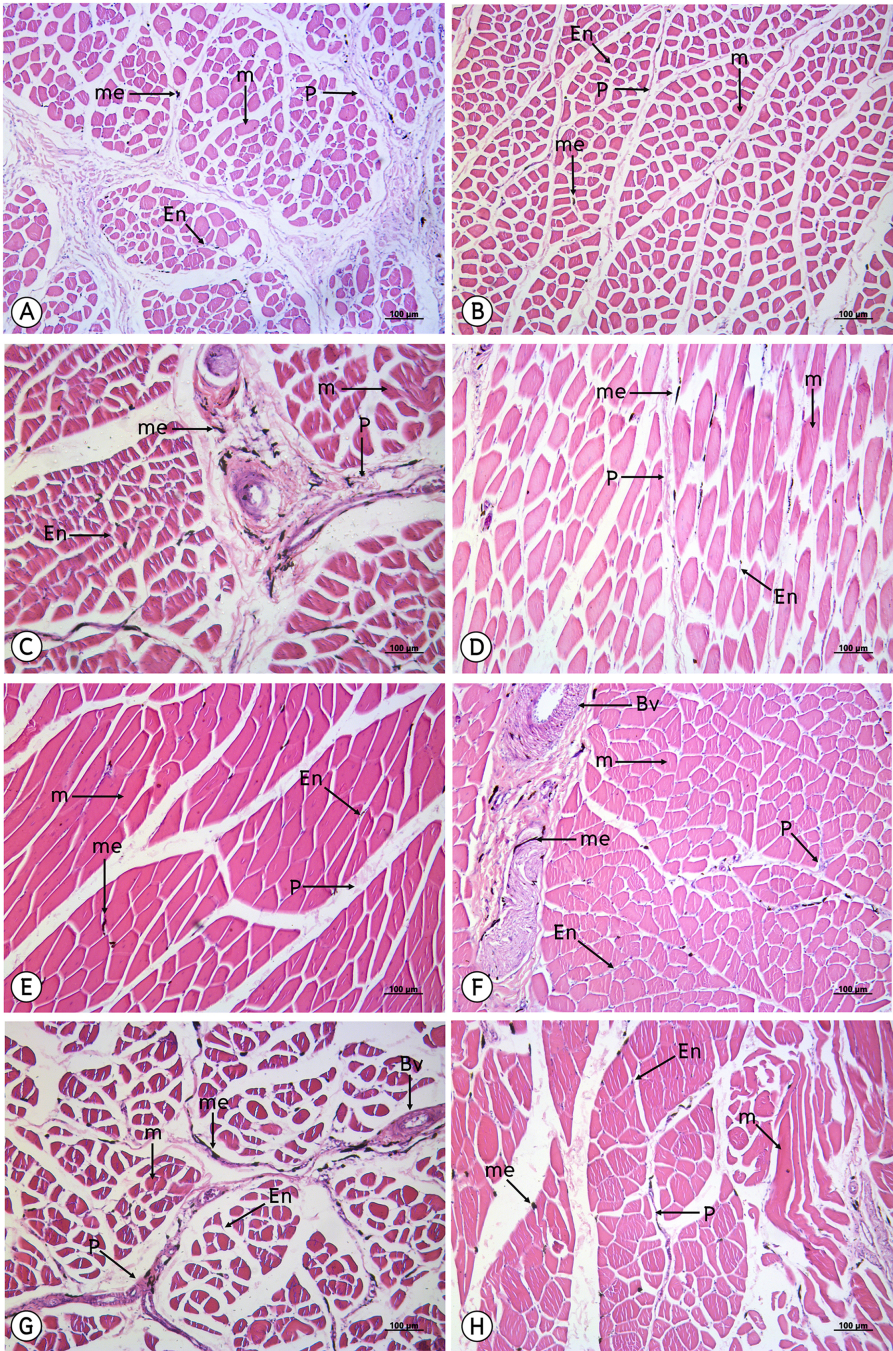
Low magnification of histological results of striated muscle from iliotibialis cranialis (A), iliotibialis lateralis pars preacetabularis (B), iliotibialis lateralis pars postacetabularis (C), flexor cruris lateralis (D), gastrocnemius (E), flexor perforans et perforatus digiti II (F), flexor perforans et perforatus digiti III (G) and fibularis longus (H). Melanin pigment accumulated in the epimysium, perimysium and endomysium, but did not accumulate in muscle fiber. Abbreviations: Bv, blood vessel; En, endomysium; M, muscle fiber; me, melanin pigment; P, perimysium. Hematoxylin and eosin staining.

**Figure 7:**
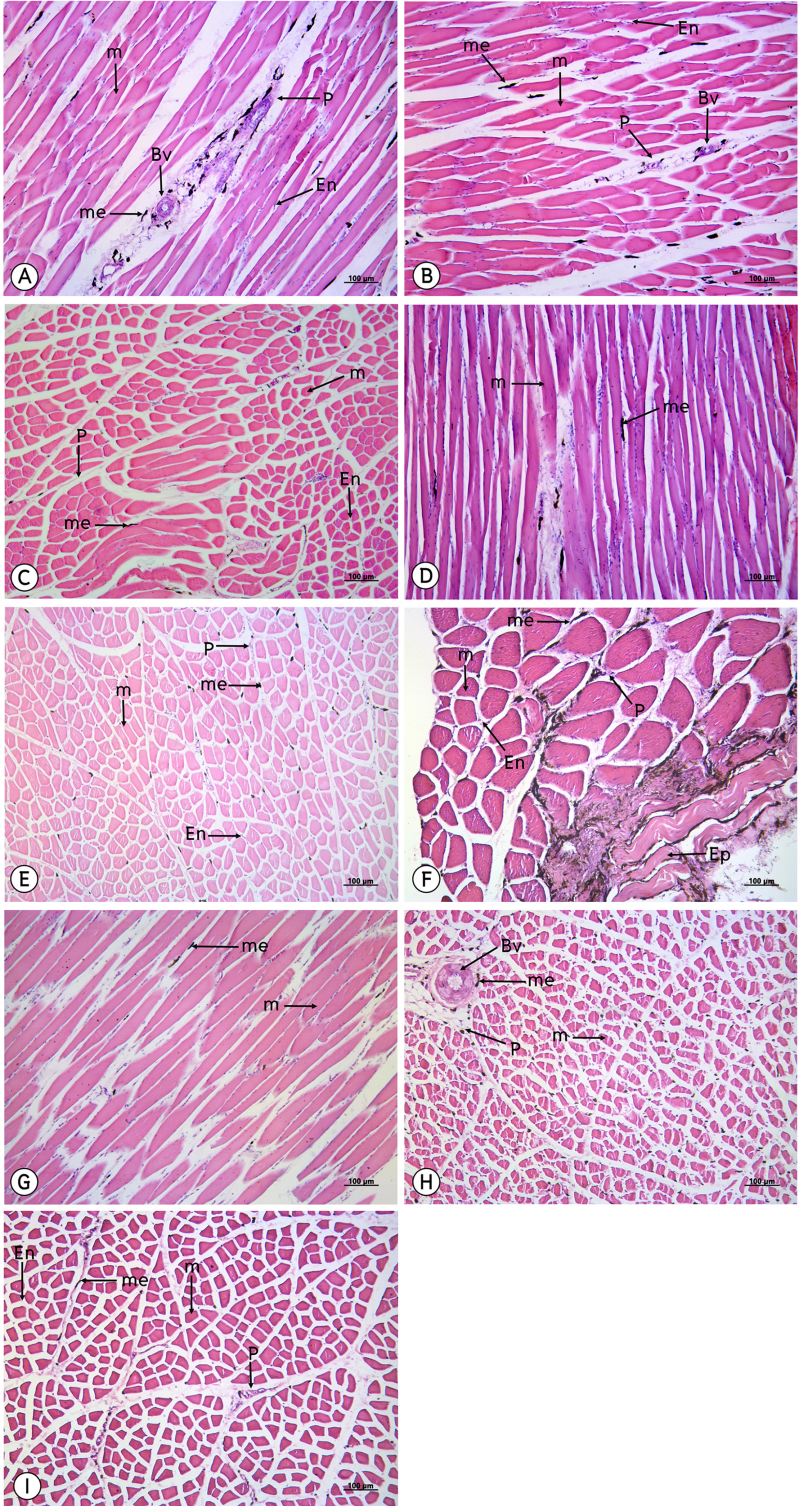
Low magnification of histological results of striated muscles from the iliotrochantericus cranialis (A), iliotrochantericus medius (B), iliofemorarlis internus (C), femorotibailis lateralis (D), flexor cruris medialis (E), puboischiofemoralis pars medialis (F), femorotibailis medialis (G), ambiens (H) and femorotibialis intermedius (I). Melanin pigment accumulated in the epimysium, perimysium and endomysium, but did not accumulate in the muscle fiber. Abbreviations: Bv, blood vessel; En, endomysium; M, muscle fiber; me, melanin pigment; P, perimysium. Hematoxylin and eosin staining.

From a study of 34 muscles, the melanin pigment was located on the epimysium, perimysium and endomysium, but did not accumulate on the muscle fiber. (Figs. 4–7).

Tthe quartile system was used for grading muscle darkness and the 34 muscles were separated into 4 groups (Table 2). In the statistical analysis of 595 muscle pairs (Fig. S1), 62 muscles displayed significant differences (*P* < 0.05), of which the highest mean percentage of melanin pigment was obtained from the flexor carpi ulnaris muscle, 1.205 ± 1.481, and the lowest percentage was obtained from the pectoralis muscle at 0.103 ± 0.072. However, there were no significant differences (*P* > 0.05) identified between the sexes (Table 3). The highest percentages of melanin pigment were found in the flexor carpi ulnaris, flexor perforans et perforatus digiti III, flexor perforans et perforatus digiti II and deltoidius pars propatagialis muscles, respectively. And the lowest percentages of melanin pigment were found in the pectoralis, iliotibialis cranialis, femorotibailis lateralis and deltoidius pars major muscles, respectively.

**Table 2:** Groups of muscle grading results based on degree of muscle darkness from the percentages of melanin pigment (mean values).

Black color	Dark grey color
Complexus (5)	Extensor carpi ulnaris (11)
Deltoidius pars propatagialis (8)	Flexor cruris medialis (30)
Femorotibailis medialis (32)	Flexor digitorum profundus (16)
Fibularis longus (25)	Iliotrochantericus cranialis (26)
Flexor carpi ulnaris (15)	Iliotrochantericus medius (27)
Flexor perforans et perforatus digiti II (23)	Latissimus dorsi (1)
Flexor perforans et perforatus digiti III (24)	Longus colli ventralis (7)
Gastrocnemius (22)	
Iliotibialis lateralis pars preacetabularis (19)	
Obliquus externus abdominis (4)	
Puboischiofemoralis pars medialis (31)	
	
**Grey color**	**Light grey color**
Ambiens (33)	Deltoidius pars major (12)
Biceps brachii (14)	Femorotibialis intermedius (34)
Biventer cervicis (6)	Femorotibailis lateralis (29)
Ectepicondyloulnaris (10)	Flexor cruris lateralis (21)
Extensor carpi radialis (9)	Iliotibialis cranialis (18)
Iliofemorarlis internus (28)	Pectoralis (3)
Iliotibialis lateralis pars postacetabularis (20)	Serratus profundus (2)
Pronator superficialis (17)	Triceps brachii (13)
	

**Table 3:** Percentages of melanin pigment between male and female chickens.

Number	Muscle name	Mean ± SD		*P* value*
		Male	Female	
1	Latissimus dorsi	0.535 ± 0.166	0.673 ± 0.445	0.596
2	Serratus profundus	0.250 ± 0.121	0.447 ± 0.119	0.091
3	Pectoralis	0.091 ±0.046	0.122 ± 0.114	0.687
4	Obliquus externus abdominis	0.700 ± 0.414	1.148 ± 0.321	0.094
5	Complexus	0.799 ± 0.672	1.185 ± 0.597	0.365
6	Biventer cervicis	0.220 ± 0.159	0.711 ± 0.437	0.065
7	Longus colli ventralis	0.497 ± 0.228	0.771 ± 0.066	0.054
8	Deltoidius pars propatagialis	0.318 ± 0.122	1.717 ± 2.336	0.317
9	Extensor carpi radialis	0.515 ± 0.215	0.468 ± 0.239	0.770
10	Ectepicondyloulnaris	0.582 ± 0.607	0.323 ±0.103	0.398
11	Extensor carpi ulnaris	0.428 ± 0.300	0.561 ± 0.398	0.569
12	Deltoidius pars major	0.183 ± 0.057	0.285 ± 0.106	0.158
13	Triceps brachii	0.245 ± 0.135	0.279 ± 0.159	0.742
14	Biceps brachii	0.489 ± 0.305	0.492 ± 0.295	0.987
15	Flexor carpi ulnaris	1.443 ± 1.743	0.612 ± 0.106	0.348
16	Flexor digitorum profundus	0.316 ± 0.140	0.782 ± 0.875	0.366
17	Pronator superficialis	0.377 ± 0.227	0.454 ± 0.329	0.716
18	Iliotibialis cranialis	0.206 ± 0.151	0.173 ± 0.028	0.653
19	Iliotibialis lateralis pars preacetabularis	0.549 ± 0.391	0.401 ± 0.324	0.553
20	Iliotibialis lateralis pars postacetabularis	0.335 ± 0.171	0.399 ± 0.185	0.604
21	Flexor cruris lateralis	0.295 ± 0.153	0.334 ± 0.145	0.751
22	Gastrocnemius	0.439 ± 0.304	0.586 ± 0.494	0.621
23	Flexor perforans et perforatus digiti II	1.430 ± 1.741	0.822 ± 0.735	0.550
24	Flexor perforans et perforatus digiti III	1.342 ± 1.209	0.852 ± 0.101	0.478
25	Fibularis longus	0.819 ± 0.575	0.575 ± 0.476	0.566
26	Iliotrochantericus cranialis	0.735 ± 0.481	0.452 ± 0.262	0.353
27	Iliotrochantericus medius	0.662 ± 0.263	0.617 ± 0.150	0.808
28	Iliofemorarlis internus	0.345 ± 0.333	0.459 ± 0.097	0.617
29	Femorotibailis lateralis	0.239 ± 0.091	0.225 ± 0.153	0.878
30	Flexor cruris medialis	0.703 ± 0.596	0.412 ± 0.299	0.499
31	Puboischiofemoralis pars medialis	0.818 ± 0.511	0.812 ± 0.622	0.989
32	Femorotibailis medialis	0.609 ± 0.161	0.711 ± 0.266	0.557
33	Ambiens	0.339 ± 0.075	0.511 ± 0.307	0.347
34	Femorotibialis intermedius	0.398 ± 0.332	0.268 ± 0.106	0.498

**Note:**

* *P* value indicates the statistical differences in mean values between male and female chickens when *p* < 0.05.

## Discussion

In the northern area of Thailand, black-bone chickens are widely reared in the highlands. Similar to other indigenous chicken breeds, variations of phenotypes, which are considered qualitative and quantitative traits, can be observed in Thai black-bone chicken populations (Buranawit et al., 2016). A variety of different black-bone breeds have emerged because the breeding area is comprised of a vast territory, diverse environments, different selections and a range of geographic proximities. Among the beneficial applications of black bone chickens is that they have been used in the treatment of many diseases such as diabetes, anemia and postpartum disorders through the reinforcement of the body’s immune system and protection against emaciation and feebleness. This study is the first report to demonstrate that 34 muscles in Thai black-bone chickens displayed an uneven distribution of darkness.

The variations in color occurring from mutations of pigmentation genes have been studied over the past decade. Examples of this include tyrosinae-related-protein-1 (Tyrp1), tyrosinase (Tyr), Epidermal growth factor receptor (Egfr) and Keratin complex 2, gene 17 (Krt2-17) (Hoekstra, 2006). In Zhang et al. (2010), studied the polymorphism of the tyrosinase gene in different Chinese chickens and found that there were many variations of Tyr genes and that mutations of this gene could affect the color of the skin. Therefore, from the results of this study, it was determined that each muscle of the Thai black-bone chicken may vary in terms of shades of darkness, which may have been influenced by mutations in the pigmentation genes of the tyrosinase gene family (Tyr, Trp-1, Trp-2). This gene family is home to the most important genes for eumelanogenesis and are responsible for inducing the synthesis of melanin (Li et al., 2011). Thus, skin melanosis of the black-bone chicken might be affected by the interactive manifestation of these two loci. Consequently, this may differently affect melanocyte development, components of melanosomes, melanosome construction or melanin synthesis. The fibromelanosis gene (Fm) and sex-linked *Id* locus were determined to have an enormous impact on inducing melanosis and determining resultant skin-color patterns and should be expressed during the early embryo stage (Arora et al., 2011). Endothelin 3 (EDN3) gene has been suggested as a candidate gene for the Fm gene and was determined to have a supportive role to *Id* locus, which also has been identified as influencing the proliferation and differentiation of the melanoblast (McCallion & Chakravarti, 2001). Id locus reflected the abnormal migratory properties of melanocyte precursors in the embryo and has an epistatic effect on Fm, while pigmentation occurs in the presence of id+ and “Fm with id” causing hyperpigmentation (Dorshorst & Ashwell, 2009). This was maintained by the actions of Fm (EDN3) throughout the body and the disparate deposition of melanocyte across different muscles (Dorshorst, Okimoto & Ashwell, 2010). Thus, the hyperpigmentation phenotype is closely related to the sex-linked incompletely dominant inhibitor of dermal melanin (*Id/id+*) and the autosomal dominant fibromelanosis (*Fm/fm+*). According to a previous study on the sex-linked gene effect of melanin in the Silkie chicken (Li et al., 2014), it is possible that the result of hyperpigmentation in the Thai black-boned chicken can be influenced by *id+* which might be homozygous in Thai black-boned chickens. Furthermore, EDN3 can be a factor that has caused hyperpigmentation in Thai black-boned chickens because the expression of this gene in ectoderm and dermamyotome was found to cause melanoblasts to migrate dorsolaterally during early differentiation phases of the embryonic stage (Li et al., 2011).

The results from this study indicated that melanin pigment accumulated in the muscle fascia, including the epimysium, perimysium and endomysium. According to the macroanatomic results, some muscles such as the iliotibialis lateralis pars preacetabularis was scored a level of 1 in terms of darkness; however, in the microanatomic results, this same muscle scored at a level of 3 with regard to degree of darkness. This might have been because the melanin pigment in the iliotibialis lateralis pars preacetabularis accumulated to a higher degree in the epimysium than in the perimysium and endomysium. Therefore, we could see that the iliotibialis lateralis pars preacetabularis was darker than some other muscles, even though the melanin pigment count was lower. This is very important as basic data as scientists can choose the correct muscle to be representative of the darkest or lightest muscle in order to study the expression of the candidate genes. Previous studies have reported that there was not enough data indicating which muscle should be use or should not be used. Consequently, the results of those studies could not provide strong evidence as to whether the lightness in the color of muscle could be used to study the expression of the candidate genes. However, the important limitation of this study was that we did not have any methods to analyze the amounts of melanin pigment between the epimysium, perimysium and endomysium. The reason for the differing amounts is still unclear and further studies are needed on the variations of muscle fascia areas for melanin pigment accumulation. On the other hand, melanocytes might accumulate differently in each muscle and this can be caused by variations in the pigmentation phenotype that is generated. Consequently, this could alter the spatial distribution of pigmentation across the body or alter the density or distribution of pigmentation along individual muscles, which would affect the overall appearance.

The results of the study of the distribution of melanin pigment by compound light microscope with 50X magnification were in accordance with those of the study conducted by Makita & Mochizuki (1984), which found that the melanin pigment was distributed from the muscle’s connective tissue such as the perimysium to the vessel connective tissue. In addition, melanin content is considered to be the most important indicator of meat quality in black-boned chickens. As such,and it provides consumers with their first visual impression of the product; thus, directly influencing their purchasing decisions (Lin & Chen, 2005; Tian et al., 2007). Therefore, in this study, our findings have indicated that smaller muscles such as the flexor carpi ulnaris, revealed the greatest average area of melanin pigment distribution that appears as a darker color, while larger muscle, such as the pectoralis, displayed the least average area of melanin pigment distribution. In other words, the pectoralis muscle is larger than the flexor carpi ulnaris muscle and the melanin pigment might have been more broadly distributed in the pectoralis than in the flexor carpi ulnaris. This would result in the flexor carpi ulnaris possessing a higher amount of the melanin pigment and also being darker in color than the pectoralis. However, immunohistochemistry techniques were employed to identify the location of melanocytes and the results were compared with the pigment areas of melanosome that were found inside the dendrite process of the melanocyte. This method might increase the percent of precision for a measurement of the area of melanin pigment. Moreover, from the previous study on the Silky fowl, the melanin pigment of many tissues such as the heart, liver, gizzard, cecum, trachea, muscle (pectoralis and supracoracoideus), skin, ovary or testis and periosteum of the femur were not different in comparisons made between sexes (Muroya et al., 2000), which was in accordance with the findings of this study where no differences were found between sexes in terms of melanin pigment distribution.

Methods that are commonly used to determine the amount of melanin pigment in the tissue include infrared spectrum (Muroya et al., 2000) and the XPS technique (Chen et al., 2008). However, in this study, a histological method was applied to calculate the ratio between the area of melanin pigment and the area of tissue similar with a prevision study (Nganvongpanit et al., 2020). Additionally, the histological method was employed because of its advantages, which are that it is easy to handle and can provide straight forward results on melanocyte distribution. According to the advantages mentioned above, the results from the normal histological method provided enough information for our study. Thus, our findings positively relate to the color chart grading results. Notably, muscles vary in size and shape and the distribution area of the melanin pigment may affect a variation in the degree of darkness present in each muscle. However, this would also be true in the vertebrates, where the melanin-based pigmentation is the culmination of a complex process including the inception, migration and regulation of melanocytes (Jackson, 1994). Likewise, the inward movement of Ca2+ from extracellular matrix causing the muscle contraction can result in pigment aggregation. Also, cytoskeleton filaments, melanocytes, as well as actin and microtubules, are in possession of essential elements that are involved in organelle transport (Nascimento, Roland & Gelfand, 2003). Furthermore, microtubules act as tracks for the transport of several intracellular organelles, including pigment vesicles and melanosomes, which are present in microtubule-poor regions of the cell. Additionally, pigment granules are transported along microfilaments. Therefore, pigment granules are mostly found in the muscles used for movement, which usually have a darker color than less contracted muscles such as the pectoralis.

Interestingly, the causes of the variations in color may have also occurred because of mutations in the genes, such as in the tyrosinase gene (Tyr gene) that controls the color expression of functional classes. On the other hand, there are also some limitations of the histology method, such as that it is time-consuming and the results may not represent a true value of overall melanin pigment, but rather may present the results as a percentage of melanin pigment in the specific tissue. Thus, the extraction of melanin directly from the muscle tissue may give faster results than the normal histological method and provide total values on the amounts of melanin pigment that accumulate in each muscle (Muroya et al., 2000). Additionally, the melanin pigment extraction method requires a higher budget for the necessary chemicals and the specific equipment needed for data analysis.

Because black-bone chickens have become economically valuable in Southeast Asian countries, and the consumption of black-bone chickens has increased, the overall consistency in the breeding of chickens with black meat is also required. The results of this recent study revealed inconsistencies in the degree of muscle darkness in black-bone chickens and the determinations of this study can be used to generate baseline information for further studies on the pigmentation gene expressions of each muscle. Notably, each muscle can display a range of darkness. It is necessary to fully understand this characteristic in order to achieve a level of consistency whole body muscle darkness in the breeding of black-bone chickens. This would be done by finding pigmentation gene mutations and studying how they are expressed.

## Conclusion

In conclusion, the results from the macroanatomical study were used with those of the histological method (microanatomy) and similar results were recorded. Moreover, these results revealed that 34 muscles of the black-bone chicken showed an uneven distribution of darkness due to the different accumulations of melanin pigments of each muscle; however, no differences were observed between the sexes. This study has provided vital information on the darkest muscles and the least dark muscles in Thai black-bone chickens. Thus, these findings indicate that muscle size and the distribution of melanin pigments can affect variations in muscle darkness and mutations of the genes that control color expression. Additionally, melanocytes may have also been identified as the main reason for the different colors of each muscle.

## Supplemental Information

10.7717/peerj.10728/supp-1 Supplemental Information 1 Raw data.

10.7717/peerj.10728/supp-2 Supplemental Information 2 The *P*-value of each muscle pair, 62 (yellow) from 595 pairs showing statistical differences at *P* < 0.05. The muscles are ordered from highest (15) to lowest (3) percentage of melanin pigment. The cells are colored with four different colors (according to Table 2). The highest degree of darkness is represented by the color black, while the lowest degree of darkness is presented in silver. (1 = latissimus dorsi, 2 = serratus profundus, 3 = pectoralis, 4 = obliquus externus abdominis, 5 = complexus, 6 = biventer cervicis, 7 = longus colli ventralis, 8 = deltoidius pars propatagialis, 9 = extensor carpi radialis, 10 = ectepicondyloulnaris, 11 = extensor carpi ulnaris, 12 = deltoidius pars major, 13 = triceps brachii, 14 = biceps brachii, 15 = flexor carpi ulnaris, 16 = flexor digitorum profundus, 17 = pronator superficialis, 18 = iliotibialis cranialis, 19 = iliotibialis lateralis pars preacetabularis, 20 = iliotibialis lateralis pars postacetabularis, 21 = flexor cruris lateralis, 22 = gastrocnemius, 23 = flexor perforans et perforatus digiti II, 24 = flexor perforans et perforatus digiti III, 25 = fibularis longus, 26 = iliotrochantericus cranialis, 27 = iliotrochantericus medius, 28 = iliofemorarlis internus, 29 = femorotibailis lateralis, 30 = flexor cruris medialis, 31 = puboischiofemoralis pars medialis, 32 = femorotibailis medialis, 33 = ambiens, 34 = femorotibialis intermedius).

## Additional Information and Declarations

### Data Availability

The following information was supplied regarding data availability: The raw measurements are available in the Supplemental Files.
