# Supplementary figures and images for: Consistency of dark skeletal muscles in Thai native black-bone chickens (Gallus gallus domesticus)
Source: PeerJ. 2021 Jan 13;9:e10728. doi: 10.7717/peerj.10728 (PMC7811297; doi:10.7717/peerj.10728)

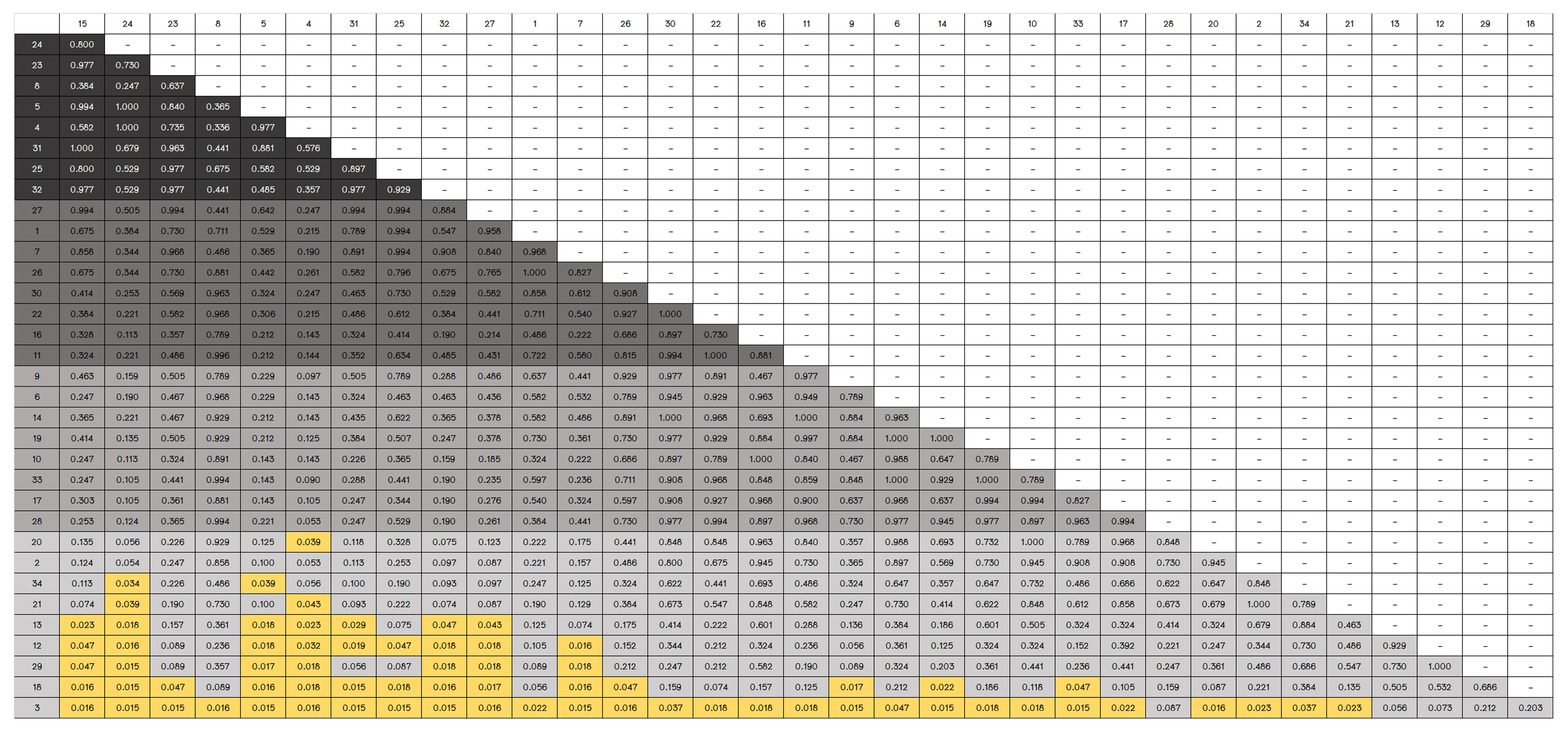

Supplement: Supplemental Information 2 — The muscles are ordered from highest (15) to lowest (3) percentage of melanin pigment. The cells are colored with four different colors (according to Table 2). The highest degree of darkness is represented by the color black, while the lowest degree of darkness is presented in silver. (1 = latissimus dorsi, 2 = serratus profundus, 3 = pectoralis, 4 = obliquus externus abdominis, 5 = complexus, 6 = biventer cervicis, 7 = longus colli ventralis, 8 = deltoidius pars propatagialis, 9 = extensor carpi radialis, 10 = ectepicondyloulnaris, 11 = extensor carpi ulnaris, 12 = deltoidius pars major, 13 = triceps brachii, 14 = biceps brachii, 15 = flexor carpi ulnaris, 16 = flexor digitorum profundus, 17 = pronator superficialis, 18 = iliotibialis cranialis, 19 = iliotibialis lateralis pars preacetabularis, 20 = iliotibialis lateralis pars postacetabularis, 21 = flexor cruris lateralis, 22 = gastrocnemius, 23 = flexor perforans et perforatus digiti II, 24 = flexor perforans et perforatus digiti III, 25 = fibularis longus, 26 = iliotrochantericus cranialis, 27 = iliotrochantericus medius, 28 = iliofemorarlis internus, 29 = femorotibailis lateralis, 30 = flexor cruris medialis, 31 = puboischiofemoralis pars medialis, 32 = femorotibailis medialis, 33 = ambiens, 34 = femorotibialis intermedius). [file peerj-09-10728-s002.png]
